# The Effects of Deep Cryogenic Treatment with Regard to the Mechanical Properties and Microstructural Evolution of Al-Mg Alloys with Different Grain Sizes

**DOI:** 10.3390/ma18194518

**Published:** 2025-09-28

**Authors:** Wei Liu, Luxiang Zhang, Erli Xia, Jing Luo, Yiran Tian, Wentao Cai, Yuqing Gong

**Affiliations:** 1 School of Intelligent Manufacturing and Mechanical Engineering, Hunan Institute of Technology, Hengyang 421002, China; 2015002039@hnit.edu.cn (W.L.);; 2 Research Institute of Automobile Parts Technology, Hunan Institute of Technology, Hengyang 421002, China; 3 School of Mechanical Engineering, University of South China, Hengyang 421001, China

**Keywords:** Al-Mg alloy, deep cryogenic treatment, grain size, mechanical properties

## Abstract

The tension behaviors of Al-Mg alloys were tested, and the influences of deep cryogenic treatment (DCT) and grain size on their tensile properties were explored. Optical microscopy (OM), scanning electron microscopy (SEM), and transmission electron microscopy (TEM) were used to characterize the evolution of the microstructure. It was concluded that the alloys with fine grain (FG) had a higher strain hardening capacity and strength, however, the alloys with coarse grain (CG) exhibited better plasticity. This can be explained by the alloy with fine grains having a higher density of grain boundary, which can hinder the motion of the dislocation; therefore, the deformation resistance was improved. For alloys with coarse grains, the dislocation has more freedom to move and is easier to rearrange, which is beneficial to the plasticity. Moreover, when given deep cryogenic treatment, the strength and plasticity of the alloys can be slightly improved, which can be attributed to the microplastic deformation that occurs during cryogenic treatment that can induce internal stress, as cold-induced internal stress is conductive in achieving a finer grain and higher density of dislocation.

## 1. Introduction

As energy issues and environmental pollution problems become more and more prominent, energy conservation and weight reduction have turned into pressing issues that need to be urgently tackled in the automotive, aerospace, and other industrial sectors [1,2,3]. Aluminum alloys, being among the lightest metallic structural materials, have seen their development and application emerge as an effective solution to address the aforementioned problems [4,5,6]. Al-Mg alloys, a typical type of aluminum alloy with extensive applications across various fields, boast a remarkable strength-to-weight ratio, outstanding formability, significant weight reduction, and excellent corrosion resistance [7,8,9]. However, with the growing demand for lightweight materials, the relatively low strength and limited ductility of Al-Mg alloys have become key bottlenecks restricting their further application. Consequently, numerous studies have been carried out by materials scientists to enhance the serving performance of Al-Mg alloys, with various methods being employed and investigated [10,11,12].

Grain size boundary strengthening is a popular way to enhance the mechanical properties of aluminum alloys. Wang et al. [13] produced commercially pure Al with different grain sizes (0.7–30 μm), where it was found that the yield strength of the aluminum increased as the grain size turned from coarse grain to fine grain, so the grain size was a deciding affect for the mechanical response during tensile deformation. Shou et al. [14] explored the effect of grain size on the tensile properties of a 2524-T3 aluminum alloy and found that the alloy’s tensile and yield strengths exhibited an inverse relationship with grain size, where smaller grains generally corresponded to increased strength values. This strength differential arises primarily from grain boundary effects on dislocation motion. Specifically, the increased density of grain boundaries in fine-grained microstructures creates additional barriers to dislocation motion; as a result, the mechanical strength of the material is enhanced. He et al. [15] investigated the hot deformation response of Al-Zn-Mg-Cu alloys that had varying grain sizes, and the results showed that the peak stress increased with the decreasing grain size. The grain size strengthening is caused by the grain boundaries, which act as obstacles during the slip of dislocations. For polycrystalline materials, the grain size strengthening can be expressed by the Hall–Petch relation [16,17,18]. The yield strength ($\sigma_{YS}$) is related to the average grain size (d) by the equation:

(1) $$\sigma_{YS}=\sigma_{0}+{\mathrm{kd}}^{-1/2}$$

where *σ*_0_ is the frictional stress resisting the glide of dislocations and *k* is a constant. So far, the effect of grain size on the tensile properties of an Al-Mg alloy processed by plastic deformation and subsequent heat treatment has seldom been reported, and is an interesting point that is worth exploring.

From previous studies, it is known that the participation of cryogenic treatment technology in the preparation and processing of aluminum alloys can significantly alter the microstructure of the material [19,20,21]. Li et al. [22] found that deep cryogenic treatment (DCT) could boost the hardness, strength, and plasticity of a 6063 aluminum alloy across the board. The DCT36h sample showed the best mechanical properties: a hardness of 142 HV, tensile strength of 328 MPa, yield strength of 177 MPa, and elongation of 29.29%. These values were 34.17%, 5.62%, 11.10%, and 32.77% higher than those of the original sample, respectively. The alloy matrix microstructure experiences lattice distortion, which generates internal stress. The internal stress causes a significant increase in dislocation generation within the alloy, resulting in a higher dislocation density. Xia et al. [23] tested the mechanical properties and microstructural evolution of the 6082 alloy with three different heat treatments, and the results showed that the DCT samples exhibited the highest strength, resulting from the pin effect induced by the high density of small precipitates. Qiu et al. [24] explored the effect of DCT on the subsequent response of the 6082 alloy to artificial aging. The results indicate that both the tensile strength and elongation of the alloy first increased and then decreased with the time of cryogenic treatment, where the fine precipitate with high density was responsible to the strengthening effect, and the change in the plasticity can be attributed to the formation of subgrains. Gao et al. [25] showed that deep cryogenic treatment (DCT) could boost the hardness and toughness of the 7A99 alloy, with a yield strength of 678 MPa and a peak elongation of 14%, surpassing those of the T6-treated alloy. DCT brings about (i) grain refinement and a more uniform microstructure and (ii) lessened segregation of Zn, Mg, and Cu atoms in the matrix as well as a reduced proportion of large clusters. Thus, DCT can refine precipitates and make their distribution more uniform, enhancing the alloy’s strength and plasticity. Guo et al. [26] examined the impact of deep cryogenic treatment (DCT) with regard to the mechanical properties and microstructure of Al-Cu-Mg-Ag alloys. Their findings revealed that DCT significantly facilitates the formation and development of the Ω phase, leading to optimized alloy properties. Additionally, DCT induces dislocation formation, further enhancing the alloy’s mechanical performance. After undergoing 1 h of DCT, the mechanical properties of the Al-Cu-Mg-Ag alloy showed great potential: its hardness reached 157 HV, tensile strength was 513 MPa, yield strength was 472 MPa, and elongation was 11.6%. Overall, deep cryogenic treatment induces lattice deformation within the alloy matrix. This process generates a significant number of dislocations, thereby increasing the dislocation density. At the same time, deep cryogenic treatment refines the grains of the alloy. All of these changes contribute to enhancing the alloy’s mechanical properties. Up to now, research on cryogenic treatment has mainly focused on 6-series and 7-series aluminum alloys. There have been very few reports about ways in which to improve the comprehensive performance of Al-Mg alloys by deep cryogenic treatment. Therefore, it is of great significance to investigate the strengthening mechanism of Al-Mg alloys processed by deep cryogenic treatment.

The Al-Mg alloy is an aluminum alloy that mainly incorporates magnesium. It has the characteristics of low density, high strength, good corrosion resistance, and excellent processing performance. It plays a key role in industrial production and daily life, and its applications cover multiple fields. Its high strength and lightweight characteristics can reduce the weight of the aircraft and improve fuel efficiency. By reducing the overall weight of the vehicle and improving alloy strength, the energy efficiency and driving performance can be improved while enhancing the driving safety and comfort. Al-Mg alloys have become a key material in industrial fields such as aerospace, automotive, and shipbuilding due to their outstanding properties. In the future, with the advancement of green manufacturing and lightweighting trends, their application potential needs to be further expanded. Previous works have demonstrated that grain size and cryogenic treatment can enhance the properties of aluminum alloys significantly, however, for Al-Mg alloys, the comprehensive effects of grain size, cryogenic treatment, and artificial aging on their enhancement and the corresponding mechanisms need further exploration. Therefore, in the present study, an Al-Mg alloy with different grain sizes was achieved through different solution processes, then, the cryogenic treatment was conducted with liquid nitrogen for different times before artificial aging. Analysis of the relationship between the microstructure and mechanical properties of the alloy was conducted through OM, SEM, and TEM, and the enhancement mechanism of the alloy material’s strength and plasticity was also explored.

## 2. Materials and Experimental Methods

The experiment alloy was a commercial 5XXX-series aluminum alloy. The chemical composition was measured, and the results are shown in Table 1.

**Figure 1:**
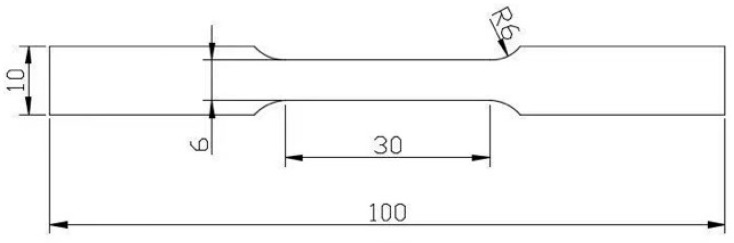
Schematic diagram of the tensile test dimensions and sampling (unit: mm).

The rolled Al-Mg alloy sheet was processed by wire-cutting to make a dog-bone specimen with a total length of 100 mm, width of 10 mm, and thickness of 3 mm, which is convenient for mechanical testing, as shown in Figure 1.

**Figure 2:**
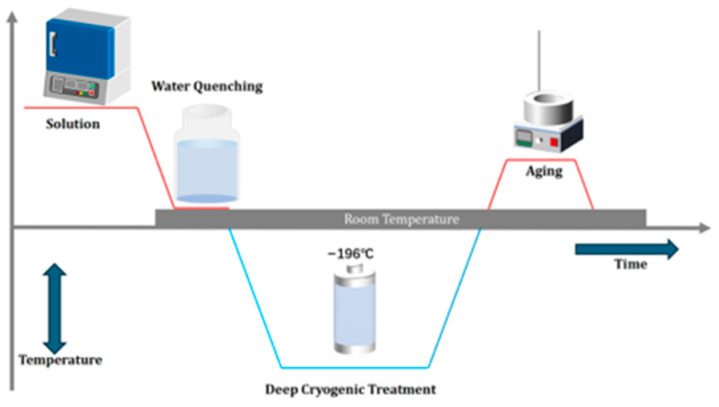
Schematic diagram of the tensile test dimensions and sampling.

**Figure 3:**
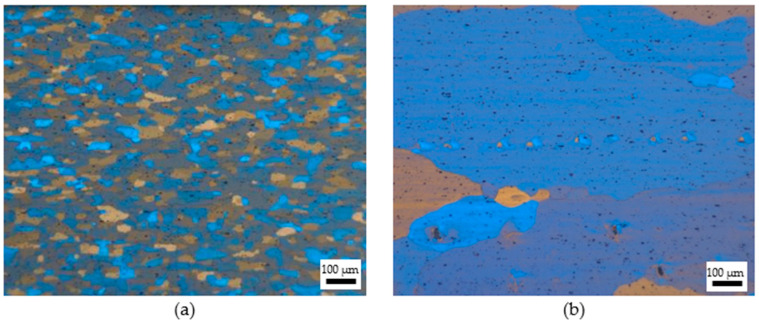
The grain structure of the specimen with different grain sizes. (**a**) FG ST; (**b**) CG ST.

The solution treatment (ST), deep cryogenic treatment (DCT), and artificial aging (AT) treatment were carried out in a resistance furnace, cryogenic treatment container, and oil bath furnace, respectively, as shown in Figure 2. The heat treatment processes of the specimen are presented in Table 2. Different solution temperatures and solution times were chosen to control the grain size of the alloy to improve the strength and ductility of the alloy. The samples were grouped into two categories. The first group of specimens were solid solution treated at 550 °C/1 h, which is a regular process to dissolve the second phase particles in the alloy and achieve a supersaturated solid solution. As shown in Figure 3a, after the solution treatment, an equiaxed grain with average size of 10 μm was obtained. The specimens in this group were referred to as the fine grain (FG) specimens. Then, parts of the FG specimens underwent DCT at −196 °C for 0–24 h. Finally, artificial aging treatment was adopted at 180 °C/8 h for all specimens. To avoid confusion, the specimens treated by ST were named FG ST, and after artificial aging treatment, the specimens was named FS DCT 0, FG DCT 4 h, and FG DCT 24 h. The second set of specimens underwent solid solution treatment at 580 °C for 2 h. It can be observed from Figure 3b that the grains became larger, with an average size of 100 µm. The specimens in this group were referred to as the coarse grain (CG) specimens. Then, the same treatment was adopted for the CG specimens, and the specimens were named CG ST, CG DCT 0, CG DCT 4 h, and CG DCT 24 h. Three samples were prepared for tensile testing under each cryogenic treatment condition. Finally, artificial aging treatment took place at 180 °C/8 h. Then, the surface of each sample was polished with sandpaper (#2000) to obtain a smooth surface. Subsequent tensile tests were conducted using an Instron 3369 universal testing machine (Instron, Norwood, MA, USA) at a strain rate of 1 × 10^−3^ s^−1^. For all conditions, three effective experiments were conducted, and the material’s stress–strain curves were derived from averaging these three datasets.

**Figure 4:**
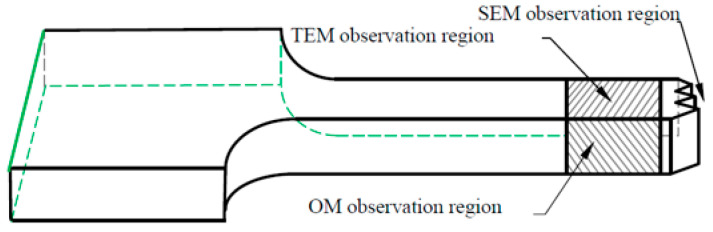
Schematic diagram of the specimen characterization area [23].

Figure 4 shows the schematic diagram of the specimen characterization area. Optical microscopy was used to reveal the microstructure prior to testing and examine the lateral surface near the fracture edge of the tested samples. The specimen was ground with 600, 1000, and 2000 grit sandpaper in turn. The polished sample was employed to achieve a scratch-free surface. The cleanliness of the sample surface was ensured through ultrasonic cleaning and cold air drying. Then, the surface was etched in a solution of 4% HBF_4_ (Runfeng Petrochemical Co., Ltd., Nantong, China) and 96% H_2_O at 20 V for 1–3 min. The specimen was observed, and the OM results were captured using an AX10 Zeiss metallographic microscope (Carl Zeiss, Oberkochen, Germany). It should be noticed that the polariscope was used to obtain the grain field contrast. SEM was used to observe the fracture surface of the tested samples. The specimen was alcohol-cleaned before SEM analysis, and the fracture morphology was observed with an EVO-18 scanning electron microscope (Carl Zeiss, Oberkochen, Germany). TEM was adopted to characterize the microstructure. TEM samples were mechanically ground to 500 µm with 600 grit sandpaper, then polished with 2000 grit sandpaper to reduce the thickness to 80 µm. Next, the specimen underwent electro-polishing in a double-jet unit at 10 V and −25 °C with a 30% nitric acid and 70% methanol solution for approximately 15 s. TEM imaging was conducted using a Tecnai G2 20 (FEI, Hillsboro, OR, USA) at 200 kV.

## 3. Results and Discussion

### 3.1. Mechanical Properties

**Figure 5:**
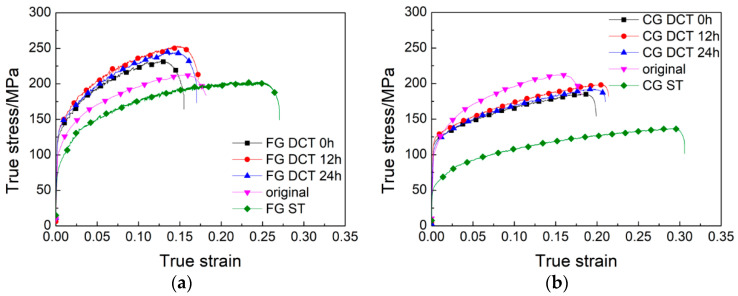
True stress–strain curves of the Al-Mg alloy with varying grain structures. (**a**) FG alloys; (**b**) CG alloys.

**Figure 6:**
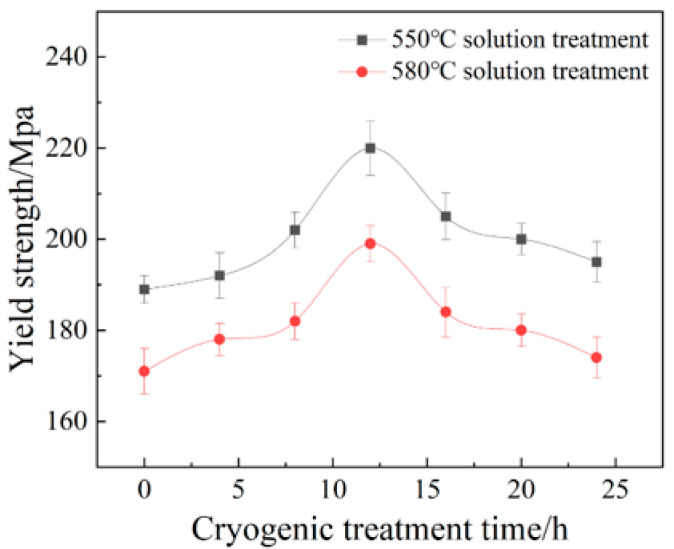
Yield strength of specimens with varying DCT times.

Figure 5 illustrates the true stress–strain curves of the Al-Mg alloys with varying grain sizes during tensile testing at room temperature. As can be seen from the figure, the deformation was initially elastic, with the material’s stress increasing rapidly with increasing strain until the yield point was reached. Upon entering the yield stage, the material underwent plastic deformation and exhibited significant strain hardening. The strain hardening phenomenon was more pronounced in the fine-grained materials. As the strain increased, the material’s strength continued to rise until the tensile strength was reached, after which fracture failure occurred. The Al-Mg alloy exhibited relatively low strength but good ductility. The yield strength of the original sample was approximately 100 MPa, showing an elongation of 17%. However, after solution treatment, the strength of the sample decreased, while its elongation rate significantly increased; for samples with coarse grains, their elongations exceeded 30%. For FG alloys (Figure 5a), the yield strength was higher than 135 MPa, which was significantly higher than the original sample, and the elongation was greater than 15%, which was close to the original sample. For CG alloys (Figure 5b), the tensile strength was lower than 125 MPa, which was slightly higher than the original sample, and the elongation was greater than 20%, which was slightly better than the original sample. For samples of the same grain type, those subjected to 12 h of deep cryogenic treatment had relatively higher strength and ductility. Figure 6 shows the yield strength of specimens with varying DCT times. The yield strength increased before 12 h, where it decreased with increasing DCT times. Within 12 h of DCT, on the one hand, a large number of supersaturated vacancies were effectively locked within the matrix, which contributed to the precipitation during the artificial aging process, resulting in the increase in yield strength. On the other hand, internal stress would be generated during DCT, which can also increase the yield strength. With a longer DCT time, the number of supersaturated vacancies decreased gradually and the internal stress became stable; the above two aspects led to the decrease in yield strength. Fine-grained materials have a higher density of grain boundaries, and dislocations are frequently impeded during slip, resulting in more pronounced dislocation pile-up and thus more significant work hardening [27,28]. Additionally, due to the impeded dislocation movement, stress concentration is more likely to occur within the material, leading to crack initiation and earlier fracture, which results in lower elongation. In contrast, coarse-grained materials have fewer grain boundaries, allowing dislocations to slip more easily over longer distances, resulting in more coordinated plastic deformation that effectively hinders crack propagation and thus permits greater plastic deformation [25]. As is known to all, there is a significant correlation between grain size and material strength. According to Equation (1), the larger the grain size, the less the yield strength. From Figure 3b, it can be concluded that the grain size of the CG alloys was much coarser, which contributed to the decrease in yield strength, as shown in Figure 5b.

### 3.2. The Characterization of Microstructure

#### 3.2.1. The Fracture Behavior

**Figure 7:**
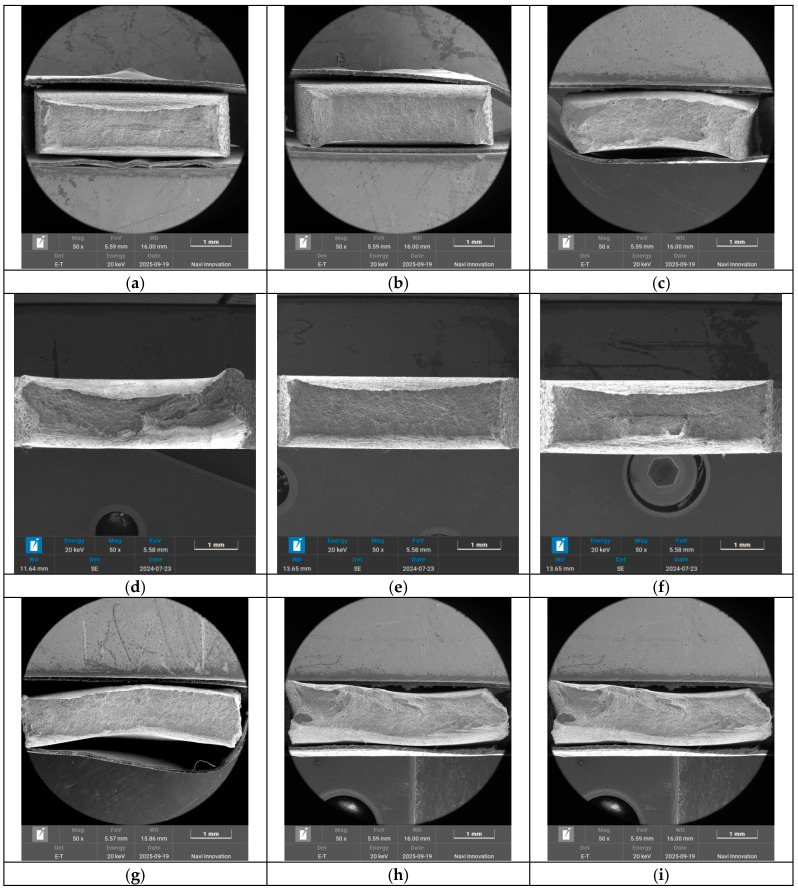
Macroscopic fracture morphology of the Al-Mg alloy sheet after different heat treatments. (**a**) Original; (**b**) FG ST; (**c**) CG ST; (**d**) FG DCT 0; (**e**) FG DCT 12 h; (**f**) FG DCT 24 h; (**g**) CG DCT 0; (**h**) CG DCT 12 h; (**i**) CG DCT 24 h.

Figure 7 shows the macroscopic fracture surfaces of the Al-Mg aluminum alloy samples after different heat treatments, all of which exhibited characteristics of ductile fracture. As can be seen from Figure 7a, the original sample exhibited good ductility, with a certain degree of necking. From Figure 7b,c, it is clear that following the solution treatment, the cross-sectional area of the fracture surface reduced significantly, reflecting superior ductility. As can be seen from Figure 7d–f, the samples with small grain sizes that underwent solution treatment at 550 °C for 1 h showed significant necking, but the cross-sectional areas of the fracture surfaces were not significantly different, indicating that the elongation of the samples was similar. Figure 7g–i reveal that the samples with large grain sizes that underwent solution treatment at 580 °C for 1 h had smaller cross-sectional areas of the fracture surfaces compared with those with small grain sizes, and the necking was more pronounced, indicating greater elongation. In addition, the samples that were not subjected to deep cryogenic treatment exhibited distortion at the fracture surface, while those that underwent deep cryogenic treatment showed better uniform deformation. It can be concluded that the specimens that underwent deep cryogenic treatment showed uniform deformation during the tensile test.

**Figure 8:**
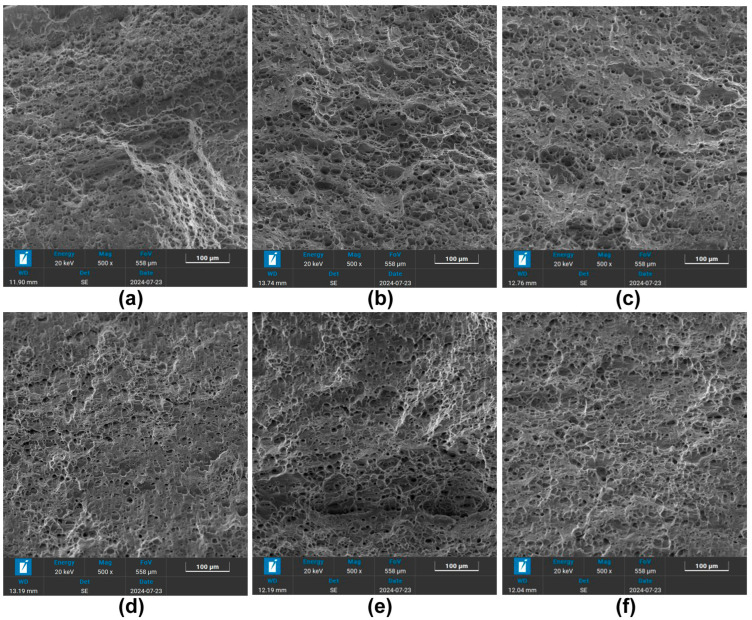
Microscopic fracture morphology of the Al-Mg alloy sheet after different heat treatments. (**a**) FG DCT 0; (**b**) FG DCT 12 h; (**c**) FG DCT 24 h; (**d**) CG DCT 0; (**e**) CG DCT 12 h; (**f**) CG DCT 24 h.

Figure 8 shows the micrographs of the fracture surfaces of the Al-Mg aluminum alloy samples after different heat treatments. The tensile fracture surfaces were all dimpled, indicating that the fracture mode was ductile fracture. As can be seen from Figure 8a–c, the small grain-sized samples treated with deep cryogenic treatment for 12 h had more and deeper dimples on the fracture surface, indicating better ductility. For the small grain-sized samples that were not treated with deep cryogenic treatment or were treated for 24 h, a large amount of planar fracture was observed, with relatively shallow dimples. From Figure 8d–f, it is evident that the large-grain-sized samples treated with solution treatment at 580 °C for 1 h had a significantly higher number of dimples and deeper dimples compared with the small grain-sized samples, indicating improved ductility of the alloy. Among them, the large grain-sized samples treated with deep cryogenic treatment for 12 h had denser and deeper dimples, showing better ductility. It can be concluded that the CG alloys showed dense and deep dimples, which indicates that a large grain contributes to better ductility.

#### 3.2.2. The Grain Structure

**Figure 9:**
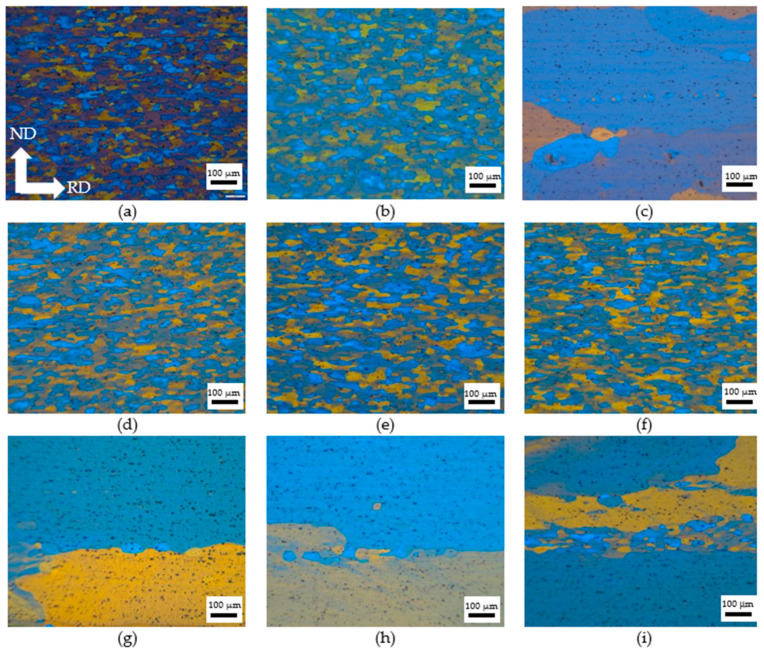
The grain structure of the specimen with varying heat treatments. (**a**) Original; (**b**) FG ST; (**c**) CG ST; (**d**) FG DCT 0; (**e**) FG DCT 12 h; (**f**) FG DCT 24 h; (**g**) CG DCT 0; (**h**) CG DCT 12 h; (**i**) CG DCT 24 h.

Figure 9 shows the micrographs of the original Al-Mg alloys with different grain sizes after various heat treatments. As displayed in Figure 9a, the original sample had fibrous grains due to the rolling process, and the average width of the grains was about 36 um. After solution treatment at 550 °C for 1 h, the grains became coarser, which showed a uniform structure with a 42 μm average grain size. As for the samples treated at 580 °C for 2 h, they showed coarse grains, and the average grain size was larger than 100 μm, while a small number of smaller grains were distributed along the grain boundaries. It is worth noting that small grains appeared along the boundaries of coarse grains, and the number of small grains increased with DCT time. For the FG alloys, the effect of DCT time on microstructure was not apparent; all specimens showed elongated grains with an average grain width of around 42 μm. For the CG alloys, all specimens showed coarse grains with a grain size larger than 100 μm. However, it should be noted that small grains existed along the grain boundary, and the number of small grains increased as the DCT time increased. The effect of DCT on grain size was not obvious, while it had a significant impact on the formation of precipitation. For the CG alloys, the longer DCT time (less than 12 h) promoted the precipitation of second phase particles during the artificial aging process. These particles would hinder the movement of dislocation, which increases the yield strength. When the DCT time increases to more than 12 h, partial dislocation may combine and disappear with a long DCT time, which would not promote the precipitation of second phase particles during the artificial aging process. The precipitation becomes nonuniform with a large size, which would decrease the yield strength.

**Figure 10:**
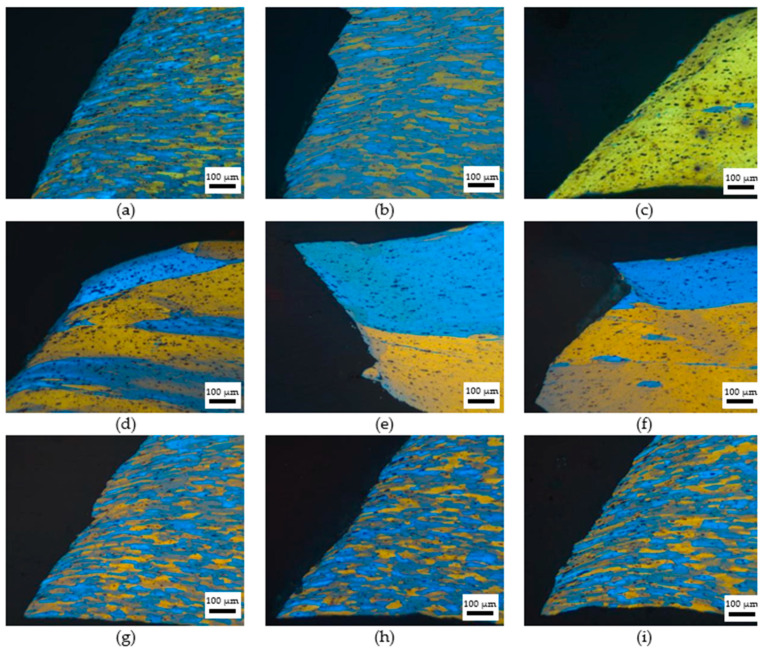
The grain structure of specimen after the tensile test. (**a**) Original; (**b**) FG ST; (**c**) CG ST; (**d**) FG DCT 0; (**e**) FG DCT 12 h; (**f**) FG DCT 24 h; (**g**) CG DCT 0; (**h**) CG DCT 12 h; (**i**) CG DCT 24 h.

Figure 10 shows the micrographs of the Al-Mg alloys with different grain sizes after tensile fracture. As can be seen from the figure, in the tensile process, grains close to the fracture surface experienced shearing and deformation along the 45° direction, and the specimen essentially failed by fracturing along the 45° direction. From Figure 9d–f, it can be observed that the alloy materials with large grains had a narrower fracture width and more pronounced necking of the specimen. For coarse-grained materials, due to the lower density of grain boundaries, dislocations can move more freely and are more easily reorganized within the grains, enhancing the dynamic recovery capability and resulting in more coordinated plastic deformation. During the tensile process, once the deformation hits a specific point, microvoids form and expand inside the alloy. Then, neighboring voids merge, eventually causing the tensile specimen to fracture. Compared with the original alloys, the ST alloys were a single supersaturated solid solution, and the deformation structure disappeared. During the tensile test, the number of stress concentration points was much lower, and the resistance of the movement of the dislocation became less, which contributed to longer elongation in the ST alloys.

**Figure 11:**
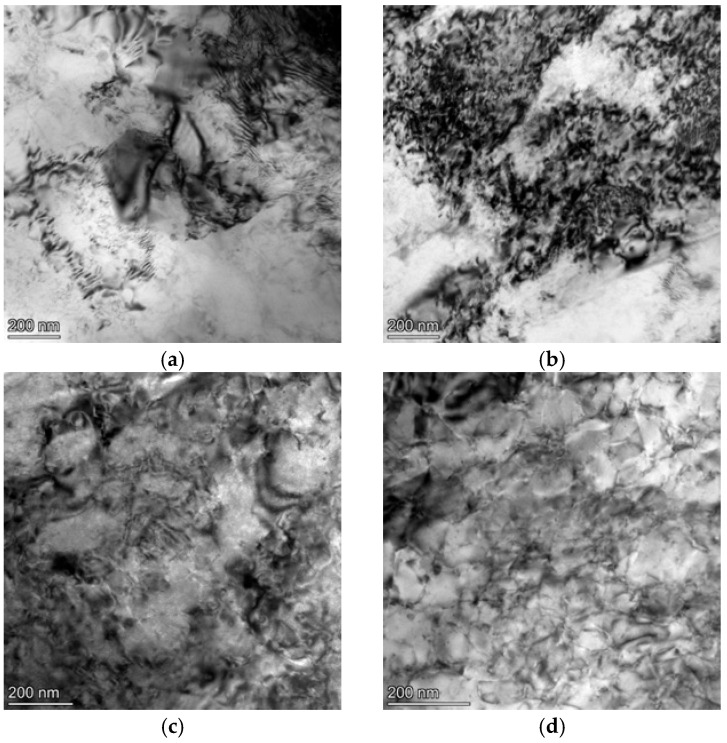

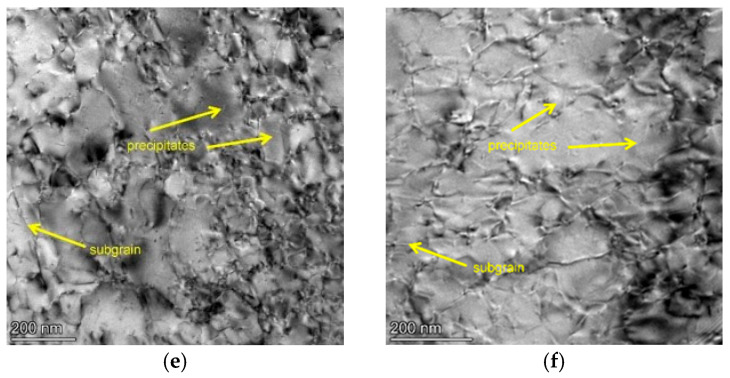
The TEM observation of the specimen after the tensile test. (**a**) FG ST; (**b**) CG ST; (**c**) FG DCT 0; (**d**) CG DCT 0; (**e**) CG DCT 12 h; (**f**) CG DCT 24 h.

The subgrain structure and dislocation distribution of the samples post-tensile fracture under different treatment conditions are illustrated in Figure 11. As illustrated in Figure 11a,b, after solution treatment, the second phase in the matrix was fully dissolved into the matrix. During the tensile process, a high quantity of dislocation structures appeared in the matrix. Since the sample with a coarse grain structure underwent greater deformation, the dislocation density in the matrix of coarse grain was higher. As seen in Figure 11c, the alloy treated with solution treatment at 550 °C for 1 h followed by aging at 180 °C for 8 h contained a large number of dislocations, but they were distributed unevenly. Figure 11d shows that in the alloy treated with solution treatment at 580 °C for 2 h followed by aging at 180 °C for 8 h, the dislocation density significantly decreased, and the distribution was relatively uniform.

It is widely recognized that the Hall–Petch relationship [29] can describe grain boundary strengthening. As can be seen from Equation (1), the samples subjected to solution treatment at 550 °C for 1 h had small grain sizes; during tensile deformation, they exhibited higher yield strength and tensile strength. This is because fine-grained materials have a higher density of grain boundaries [29,30]. During deformation, these boundaries can effectively impede dislocation movement and lead to dislocation pile-up, increasing the dislocation density and thus enhancing the resistance to deformation. At the same time, they are also more prone to stress concentration, which could cause the material to fracture.

As shown in Figure 11e,f, after 24 h of deep cryogenic treatment, the number of subgrains in the alloy increased, and the dislocation density was enhanced, with fewer dislocation tangles observed. After 12 h of deep cryogenic treatment, the dislocation density was significantly increased, with more densely packed dislocations and a greater number of dislocation tangles as well as the formation of a small number of dislocation cell structures. When the temperature was reduced from room temperature to −193 °C, the alloy underwent microplastic deformation, resulting in the generation of cold-induced internal stress. According to the equation of state for solid materials [20,31]:

(2) $$V_{T}=V_{0}e^{\alpha \;(T-T_{0})}$$

The change in volume is:

(3) $$\Delta V=V_{T}-V_{0}=V_{0}\left[e^{\alpha (T-T_{0})}-1\right]$$

In the equation, *V*_0_ is the volume of the solid at temperature *T*_0_, *V_T_* is the volume of the solid at temperature *T*, and *α* is the coefficient of expansion. The volume contraction that occurs in the alloy during deep cryogenic treatment corresponds to the volumetric strain. The average internal stress should exclude the original internal stress of the alloy and take the average of the three principal stresses. Therefore, the average internal stress experienced by the alloy can be expressed as:

(4) $$\sigma_{m}=K\theta =K\left[e^{\alpha (T-T_{0})}-1\right]$$

In the equation, K is the elastic modulus of the alloy. The internal stress within the alloy is conducive to grain refinement and increases the dislocation density. After deep cryogenic treatment, a large number of dislocations proliferate internally, resulting in an increase in dislocation density. Moreover, the increase in dislocation density in the alloy after deep cryogenic treatment leads to a significant migration of dislocations toward the grain boundaries, forming relatively dense low-energy dislocation cells near the grain boundaries. During the subsequent aging process, the dislocations at the grain boundaries rapidly diffuse. The interactions among some unstable dislocations during diffusion promote the transformation of these dislocation cells into subgrains [32], thereby enhancing the strength of the material and improving its deformation capability.

The strength of the alloy can be influenced by small precipitates in the matrix. As the size of the precipitates inside the alloy is small, dislocations are prone to cut out of the precipitate. The motion mechanism of dislocations is the cutting mechanism, and the strengthening effect can be expressed as:

(5) $$\Delta \tau =\lambda f^{1/2}\gamma^{1/2}$$

In the equation, λ represents a constant, f denotes the volume fraction of the precipitates, and γ refers to the size of the precipitates. The strength of the alloy with finer precipitates is positively correlated with the size and volume fraction of the precipitates. Comparing Figure 11e,f with Figure 11c,d, the precipitate in the matrix is more pronounced, proving that cryogenic treatment is beneficial for the formation of precipitates, which can be attributed to the higher strength of the alloy with cryogenic treatment.

Furthermore, it can be observed from the TEM images that a small number of precipitates were sporadically distributed in the matrix. Generally, the proper DCT time promotes the precipitation of second phase particles during the artificial aging process. With a longer DCT time, partial dislocation may combine and disappear, which may inhibit the precipitation of second phase particles. On the one hand, after deformation, there is a high density of dislocations in the matrix, which is not conducive to the observation of precipitates. On the other hand, the studied alloy in this work had a main element of Mg and the Si element concentration was low, so was unable to form a large number of precipitates. The limited number of precipitates is responsible for the slight improvement in strength compared with the other series of aluminum alloy.

## 4. Conclusions

Tensile testing was performed at ambient temperature in order to explore the effects of deep cryogenic aging treatments and grain size on the mechanical properties of an Al-Mg alloy. The key findings are summarized as follows:

Compared with Al-Mg alloys with coarse-grained structures, materials with fine-grained structures exhibited higher strain hardening capability and strength, but their elongation was relatively lower. For fine-grained Al-Mg alloys, the yield strength was above 135 MPa, while the elongation was greater than 15%. In contrast, for coarse-grained materials, the tensile strength was below 125 MPa, with an elongation greater than 20%. Fine-grained materials have a higher density of grain boundaries, which can effectively impede dislocation movement, leading to dislocation pile-up, and thus enhancing the material’s resistance to deformation. In contrast, dislocations in coarse-grained materials have greater freedom to move and are more easily reorganized, thereby improving the deformation capacity and delaying necking and fracture.

Deep cryogenic treatment is conducive to enhancing the strength and ductility of materials. For both coarse-grained and fine-grained Al-Mg alloys, the alloys kept at −196 °C for 12 h and subsequently subjected to artificial aging at 180 °C for 8 h showed the best mechanical properties. This is because deep cryogenic treatment induces microplastic deformation within the alloy, leading to the generation of cold-induced internal stress. The cold-induced internal stress is beneficial for grain refinement and increased dislocation density, thereby enhancing the material’s strength and ductility.

## Figures and Tables

**Table 1:** Chemical composition of the Al-Mg aluminum alloy sheet (mass fraction, %).

Element	Fe	Cu	Si	Cr	Mg	Mn	Ti	Al
wt%	0.25	0.02	0.12	0.15	2.13	0.09	0.02	Balance

**Table 2:** Heat treatment process and parameters.

Type	Solution Treatment	Deep Cryogenic Treatment	Artificial Aging
FG alloys	550 °C/1 h; water quenching	0, 4 h, 8 h, 12 h, 16 h, 20 h, 24 h	180 °C/8 h
CG alloys	580 °C/2 h; water quenching	0, 4 h, 8 h, 12 h, 16 h, 20 h, 24 h	180 °C/8 h

## Data Availability

The original contributions presented in this study are included in the article. Further inquiries can be directed to the corresponding author.
